# Characteristics of patients with heart failure with preserved ejection fraction in primary care: a cross-sectional analysis

**DOI:** 10.3399/BJGPO.2021.0094

**Published:** 2021-11-10

**Authors:** Faye Forsyth, James Brimicombe, Joseph Cheriyan, Duncan Edwards, FD Richard Hobbs, Navazh Jalaludeen, Jonathan Mant, Mark Pilling, Rebekah Schiff, Clare J Taylor, M Justin Zaman, Christi Deaton, on behalf of the OPTIMISE HFpEF investigators and collaborators

**Affiliations:** 1 Department of Public Health and Primary Care, University of Cambridge School of Clinical Medicine, Cambridge, UK; 2 Division of Experimental Medicine and Immunotherapeutics, Department of Medicine, University of Cambridge School of Clinical Medicine, Cambridge, UK; 3 Nuffield Department of Primary Care Health Sciences, University of Oxford, Oxford, UK; 4 Department of Ageing and Health, Guy’s and St. Thomas’ NHS Foundation Trust, London, UK

**Keywords:** heart failure with preserved ejection fraction, primary health care, multimorbidity, frailty

## Abstract

**Background:**

Many patients with heart failure with preserved ejection fraction (HFpEF) are undiagnosed, and UK general practice registers do not typically record heart failure (HF) subtype. Improvements in management of HFpEF is dependent on improved identification and characterisation of patients in primary care.

**Aim:**

To describe a cohort of patients recruited from primary care with suspected HFpEF and compare patients in whom HFpEF was confirmed and refuted.

**Design & setting:**

Baseline data from a longitudinal cohort study of patients with suspected HFpEF recruited from primary care in two areas of England.

**Method:**

A screening algorithm and review were used to find patients on HF registers without a record of reduced ejection fraction (EF). Baseline evaluation included cardiac, mental and physical function, clinical characteristics, and patient reported outcomes. Confirmation of HFpEF was clinically adjudicated by a cardiologist.

**Results:**

In total, 93 (61%) of 152 patients were confirmed HFpEF. The mean age of patients with HFpEF was 79 years, 46% were female, 80% had hypertension, and 37% took ≥10 medications. Patients with HFpEF were more likely to be obese, pre-frail or frail, report more dyspnoea and fatigue, were more functionally impaired, and less active than patients in whom HFpEF was refuted. Few had attended cardiac rehabilitation.

**Conclusion:**

Patients with confirmed HFpEF had frequent multimorbidity, functional impairment, frailty, and polypharmacy. Although comorbid conditions were similar between people with and without HFpEF, the former had more obesity, symptoms, and worse physical function. These findings highlight the potential to optimise wellbeing through comorbidity management, medication rationalisation, rehabilitation, and supported self-management.

## How this fits in

HFpEF is common (about half of all patients with HF) but the condition is often unrecognised and poorly managed. To the authors’ knowledge, no previous studies have provided a detailed characterisation of patients with HFpEF within primary care HF registers. This study confirmed diagnosis and phenotyped a cohort of patients recruited from primary care with possible HFpEF, comparing patients in whom HFpEF was confirmed with patients in whom HFpEF was refuted. Patients with HFpEF were differentiated from patients not meeting HFpEF diagnostic criteria by higher levels of obesity, frailty and symptoms, and worse physical functioning. Self-management and self-monitoring of worsening signs and symptoms of HF were extremely limited in patients with HFpEF. Management of comorbidities in HFpEF is essential but complex. It needs to incorporate medication reviews, and increased use of non-pharmacological interventions such as self-management support and exercise training or cardiac rehabilitation. Polypharmacy could be decreased by better differentiation between patients with HFpEF and heart failure with reduced ejection fraction (HFrEF).

## Introduction

HFpEF accounts for half of all HF and 70% of those with HF aged >65 years.^1^ Current evidence suggests HFpEF is driven by comorbid conditions, especially obesity, hypertension, diabetes, and kidney disease, leading to systemic inflammation and endothelial microvascular dysfunction.^1,2^ Despite its prevalence, HFpEF remains poorly diagnosed, managed, and researched.^3–6^ Under-recognition of HFpEF relates to lack of awareness and uncertainty regarding its pathophysiology, treatment, and diagnostic criteria. Pathways to HF diagnosis are variable, and limited knowledge of HFpEF and a lack of relevant echocardiographic information leads to under-identification in primary care.^3–5,7^

Most patients with HF are managed in primary care, especially those with HFpEF who may not be referred to specialists, or if referred not provided with a treatment plan.^3,8^ Evidence for effective pharmacological treatment specific to HFpEF is sparse. Current recommendations are to control comorbid conditions and use diuretics to manage volume overload.^9^ Lack of pharmacological treatment is thought to relate to patient heterogeneity, leading to interest in defining phenotypes that might respond to specific therapy. Phenotyping has been based on populations recruited into clinical trials with limited comorbidity or admitted for acute HF, and thus not representative of most patients in the community.^10–13^ Characterising patients with HFpEF in primary care is an essential step towards improving diagnosis and management, as well as recruiting into trials.

This analysis presents baseline data from a longitudinal observational study that is a component of the Optimising Management of Patients with Heart Failure with Preserved Ejection Fraction in Primary Care (OPTIMISE HFpEF) study.^14^ Patients were recruited based on a search of primary care HF registers for patients with no record of reduced ejection fraction. In this report, the baseline characteristics of those patients in the cohort who were confirmed as having HFpEF are described, and they are compared to patients in whom HFpEF was not confirmed.

## Method

### Study design and setting

Study participants were recruited from 30 general practices in two regions of England: East of England and Oxfordshire, Thames Valley. Practices were included from cities, towns, and semi-rural areas varying by Index of Multiple Deprivation (IMD) score from high deprivation to more affluent areas (IMD 2–9). Owing to slow accrual, patients were also recruited from an older persons’ clinic in London and a HF service in Cambridgeshire receiving primary care referrals. The study is supported by an active patient advisory group, and patients were involved in development and analysis.

### Participants

Patients with possible HFpEF were identified via an electronic medical record screening algorithm of HF registers in general practices and physical record screening in the outpatient clinics. The algorithm was designed to screen out patients with codes for left ventricular systolic dysfunction (LVSD) and cardiomyopathy. Patients identified in the electronic search were screened by GPs against study criteria. Exclusion criteria included an EF <50%, moderate to severe systolic dysfunction, significant cognitive impairment, or end-of-life care. Patients deemed eligible were invited to participate by letter from the practice. Those interested attended a baseline assessment where informed consent was obtained.

HFpEF diagnosis was clinically adjudicated by a cardiologist based on a global evaluation of the available history of any heart failure symptoms, signs of HF, consideration of natriuretic peptide levels, and evidence of relevant structural heart disease and/or diastolic dysfunction on transthoracic echocardiogram (TTE), as per European Society of Cardiology (ESC) guidelines criteria at start of recruitment (Box 1). A more detailed discussion of the diagnostic process for the study is available elsewhere.^15^

Box 1 **European Society of Cardiology criteria for diagnosis of HFpEF^9^**

- Signs and symptoms of heart failure
- Ejection fraction >50%
- Elevated natriuretic peptides:
  - NT-proBNP >125 pg/ml
  - BNP >35 pg/ml
- Evidence of relevant structural heart disease and/or diastolic dysfunction:
  - Left ventricular hypertrophy: left ventricular mass index >115 g/m^2^ for males and >95 g/m^2^ for females
  - Increased left atrial volume index: >34 ml/m^2^
  - Early diastolic tissue velocity (e’mean septal-lateral <9 cm/s)
  - Ratio between early mitral inflow velocity and mitral annular early diastolic velocity (E/e’ >13)
  - E/A ratio <1 or >2
  - Deceleration time of mitral valve early diastolic inflow m/s (normal is <240 m/s)
  - Isovolumetric relaxation time

BNP = brain natriuretic peptide. HFpEF = heart failure with preserved ejection fraction. NT-proBNP = N-terminal pro B-type natriuretic peptide.

### Variables

Variables included: physical characteristics; past medical history and comorbidities; heart function (12-lead electrocardiogram and transthoracic echocardiogram [TTE]); oedema assessment; breathlessness and fatigue (modified Borg); frailty assessment by Clinical Frailty Scale (CFS); Survey of Health, Ageing and Retirement in Europe Frailty Instrument (SHARE-FI); cognition assessment (Montreal Cognitive Assessment [MoCA]); physical functioning (6-minute walk distance, gait speed) and physical activity levels (7-day accelerometer wear); laboratory testing (biochemistry, haematology, biomarkers); anxiety and depression (Hospital Anxiety and Depression Score [HADS]); HF quality of life (Kansas City Cardiomyopathy Questionnaire [KCCQ]); HF self-care (European Heart Failure Self-care Behaviour [EHFScB] Scale); HF symptoms (Symptom Status Questionnaire — Heart Failure); and health-related quality of life (EQ-5D-5L). Validated assessments, standardised equipment, and a detailed manual of operations and procedures promoted consistency across sites.

### Sample size

The target sample size was 270 recruited, with an estimated 25% not being confirmed as HFpEF^16^ to give a sample of 200 patients. It was anticipated that 40% of patients on HF registers would be identifiable as possible HFpEF.^5^

### Statistical analysis

Patient characteristics and assessments were described using frequencies, measures of central tendency, and proportions as appropriate. Normality for continuous data were assessed using the Shapiro-Wilk test and Q-Q plots. Normally distributed data are presented as mean±standard deviation, non-parametric as median and interquartile range (25%–75%), and categorical data as absolute number and per cent. Descriptive statistics are presented for the cohort, and comparisons according to confirmed HFpEF versus non-HFpEF using χ^2^ for categorical variables and *t*-tests for normally distributed continuous data. Where data are missing, values are reported as such. Statistical analyses were performed using R (version 3.63) and IBM SPSS Statistics (version 27).

## Results

**Figure 1.**
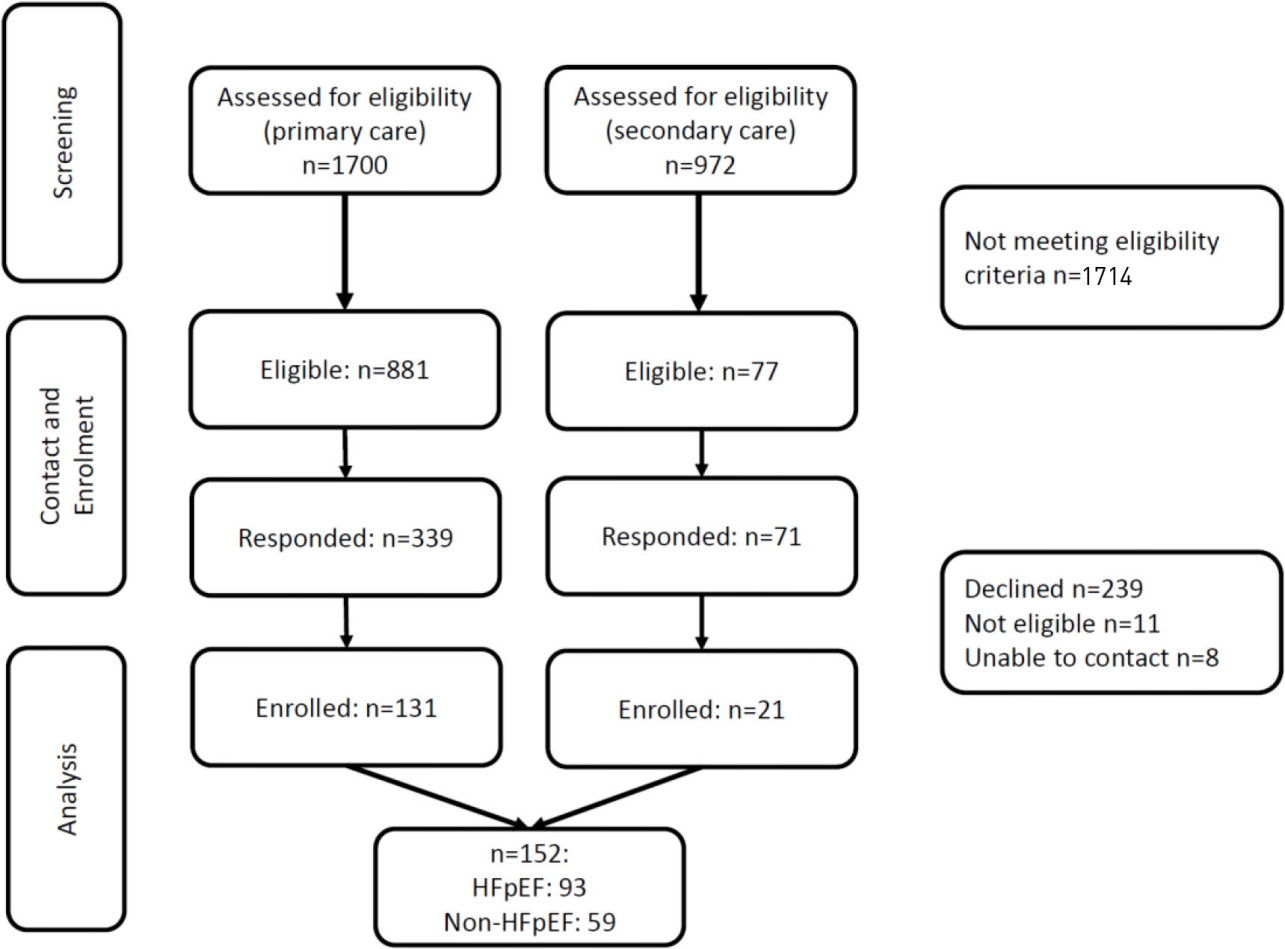
Patient flow chart. HFpFF = heart failure with preserved ejection fraction.

In primary care, 52% of patients on HF registers were considered eligible. Between July 2018 and November 2019, 152 patients were recruited, 16% of those were eligible (Figure 1). Ninety-three (61%) were clinically adjudicated to have HFpEF. Participants with HFpEF (Table 1) had a mean age of 79 years (±7.1), 46% were female, and 60% had a history of smoking. Mean Charlson Comorbidity Index (CCI) was 4.8, and the majority were overweight or obese. Functional impairment was evident by 6MWD and gait speed, and 63% had mild cognitive impairment. Over half were considered pre-frail or frail, and 40% were considered sarcopaenic by grip strength and gait speed. Sixteen per cent reported occasional incontinence, and 4% were incontinent or had indwelling catheters.

**Table 1.** Characteristics of sample and by HFpEF diagnosis

Characteristic	*n*	Total sample, *n* = 152, %^a^	Confirmed HFpEF, *n* = 93, %^a^	Non-HFpEF,*n* = 59, %^a^	*P* value forcomparison^b^
Age, years, mean (SD)	152	78 (8.6)	79 (7.1)	77 (10.5)	0.156
Sex, female	152	40	46	29	**0.039**
LVEF, mean (SD)	148	56.9 (9.2)	58.1 (7.1)	54.4 (10.8)	**0.023**
History of smoking	152	67	60	79	**0.015**
CCI, mean (SD)	150	4.6 (2.6)	4.8 (2.8)	4.2 (2.2)	0.157
Hypertension	150	80	80	77	0.636
Diabetes	150	29	32	26	0.498
Chronic lung disease	150	29	32	23	0.251
Moderate to severe kidney disease	150	33	34	32	0.789
Previous myocardial infarction	149	13	12	14	0.730
Peripheral vascular disease	150	9	7	12	0.226
Previous stroke or TIA	150	14	14	16	0.781
Cancer	150	16	14	19	0.404
BMI, mean (SD)	151	30.4 (6.6)	30.9 (6.2)	29.4 (7.1)	0.179
Overweight	151	26	25	28	0.053
Obese	151	50	57	39	
Combined overweight or obese	151	76	82	67	**0.036**
NYHA class I	152	22	17	31	0.118
NYHA class II	152	57	62	48	
NYHA class III	152	20	20	21	
Leg oedema	152	45	46	43	0.707
Sinus rhythm	152	45	50	39	0.435
Atrial fibrillation	152	34	32	39	
Other	152	21	19	23	
Heart rate, mean (SD)	145	68 (14)	68 (13)	69 (15)	0.556
SBP, mean (SD)	150	136 (23)	138 (23)	134 (22)	0.346
SBP >150, mean %	150	31	33	28	0.535
DBP, mean (SD)	150	77 (12)	77 (12)	78 (11)	0.577
DBP >90, mean %	150	16	15	18	0.643
Pulse pressure, mean (SD)	150	59 (19)	61 (17)	56 (20)	0.142
MoCA score, mean (SD)	146	25.4 (3.3)	24.9 (4.3)	24.8 (5.7)	0.951
Mild cognitive impairment	146	58	63	48	0.194
Pre-frail	148	32	36	27	0.101
Frail	148	22	26	18	
Combined pre-frail and frail	148	54	63	45	**0.033**
6-minute walk distance, mean (SD)	117	296 (127)	273 (123)	338 (125)	**0.007**
Time to walk 10 m, sec, mean (SD)	117	10.8 (6.4)	11.7 (7.4)	9.1 (3.6)	**0.014**
Gait speed, m/s, mean (SD)	117	1.13 (0.47)	1.05 (0.39)	1.3 (0.55)	**0.010**
Activity levels by median daily vector magnitude (IQR)	124	16.2 (12.2–20.2)	15.4 (12.0–18.3)	18.2 (12.9–21.5)	**0.018**
Sarcopaenia	147	35	40	29	0.176
Occasional incontinence	151	17	16	19	0.867
Incontinent or catheterised	151	4	4	4	
**Patients with known hypertension**					
SBP, mean (SD)	122	142.3 (22.2)	144.5 (22.8)	138.9 (20.9)	0.203
DBP, mean (SD)	122	79.8 (10.9)	79.3 (11.1)	80.6 (10.8)	0.529
Pulse pressure, mean (SD)	122	62.6 (18.7)	65.2 (18.6)	58.3 (18.4)	0.059

^a^Unless otherwise stated. ^b^Bold indicates statistically significant value. BMI = body mass index. CCI = Charlson Comorbidity Index. DBP = diastolic blood pressure. HFpEF = heart failure with preserved ejection fraction. IQR = interquartile range. LVEF = left ventricular ejection fraction. MoCA = Montreal Cognitive Assessment. NYHA = New York Heart Association. SBP = systolic blood pressure. SD = standard deviation. TIA = transient ischaemic attack.

Although the initial aim was to characterise and follow-up patients with HFpEF, the authors took the opportunity in this baseline analysis to compare patients confirmed as HFpEF with those not considered to have HFpEF. The non-HFpEF group primarily had a mixture of other HF diagnoses (for example, valvular heart disease, hypertrophic cardiomyopathy, and recovered EF), although the investigations were only intended to diagnose patients without HFpEF. When delineating by confirmation of HFpEF, patients with HFpEF were more likely to be pre-frail or frail, and have greater functional impairment based on 6MWD and gait speed. Patients with HFpEF were less physically active and spent more time in very low levels of activity compared with those not confirmed HFpEF.^17^ Patients with HFpEF walked 65 m less than the non-HFpEF group, and took >2 seconds longer to walk 10 m. Sarcopaenia was more prevalent in the HFpEF versus non-HFpEF group (40% versus 29%, *P* = 0.176) (Table 1).

Laboratory tests were available for 131 (86%) participants (Table 2). Values were not significantly different between groups, although an estimated glomerular filtration rate (eGFR) <30 ml/min was more frequent in those with HFpEF compared with those without. Natriuretic peptides (NT-proBNP) levels were a median of 301 pg/ml (interquartile range [IQR] 73–1029) in the HFpEF group, and 332 pg/ml (IQR 147–1112) in those without HFpEF. A small number of patients (six with HFpEF) presented with NT-proBNP levels >2000 pg/ml. Twice as many patients with HFpEF had HbA1c levels >48 mmol/l than those without HFpEF (26% versus 13%, *P* = 0.085), and mean HbA1c in 39 patients known to have diabetes was 56.4±16.7 mmol/l.

**Table 2.** Laboratory values by HFpEF diagnosis

Parameter	*n*	Total sample, *n* = 152, mean (SD)^a^	Confirmed HFpEF, *n* = 93, mean (SD)^a^	Non-HFpEF, *n* = 59, mean (SD)^a^	*P* value for comparison
NT-proBNP, pg/ml, median (IQR)	111	314 (124–1055)	301 (73–1029)	332 (147–1112)	0.841
eGFR	129	66 (21)	65 (21)	70 (20)	0.190
eGFR <30, %	129	5	8	0	0.042
Random glucose, mmol/l	120	6.8 (4)	6.9 (3)	6.7 (4)	0.797
Sodium, mmol/l	129	139 (3)	139 (3)	139 (3)	0.286
Potassium, mmol/l	128	4.2 (0.5)	4.2 (0.4)	4.2 (0.5)	0.401
Creatinine, μmol/l	130	93 (39)	95 (43)	90 (31)	0.570
Urea, mmol/l	122	8.6 (5)	8.9 (6)	8.1 (3)	0.378
Haemoglobin, g/l	131	131 (17)	130 (15.5)	135 (19)	0.130
Haematocrit, %	129	0.4 (0.04)	0.39 (0.05)	0.41 (0.06)	0.141
Platelets	130	229 (77)	232 (77)	227 (76)	0.677
HbA1c	129	45 (13)	46 (12)	43 (14)	0.250
HbA1c >48, %	129	22	26	13	0.085
HbA1c known diabetes	39	56.4 (16.7)	56.5 (14.2)	56.2 (22.6)	0.955

^a^Unless otherwise stated. eGFR = estimated glomerular filtration rate. HbA1c = glycosylated haemoglobin A1c. HFpEF = heart failure with preserved ejection fraction. NT-proBNP = N-terminal pro B-type natriuretic peptide. SD = standard deviation.

Patient reported outcome measures (Table 3) showed no statistically significant differences in scores except for daytime dyspnoea and fatigue (worse in people with HFpEF). Pharmacological treatment (Table 4) did not differ significantly between groups, with both prescribed an average of eight medications. Approximately one-third of patients were on ≥10 medications. Most patients were prescribed diuretics, and about half were on beta-blockers. In contrast to pharmacological treatment, cardiac rehabilitation was infrequent.

**Table 3.** Patient reported measures by HFpEF diagnosis

Patient reported outcome measures	HFpEF, *n* = 93, %^a^	Non-HFpEF, *n* = 59, %^a^	*P* value for comparison
**Kansas City Cardiomyopathy Questionnaire**			
Physical limitations, mean (SD)	67 (28)	73 (28)	0.205
Quality of life, mean (SD)	69 (29)	73 (25)	0.410
Symptom total, mean (SD)	72 (25)	78 (26)	0.171
Clinical summary, mean (SD)	70 (24)	77 (25)	0.118
Summary, mean (SD)	71 (25)	74 (25)	0.374
**Hospital Anxiety and Depression Scale**			
Depression mean (SD)	7.6 (2.3)	7.2 (2.5)	0.236
Moderate-to-severe depressive symptoms	8.9	6.9	0.810
Anxiety mean (SD)	5.2 (4.4)	4.7 (3.9)	0.455
Moderate-to-severe anxiety symptoms	11.2	12.1	0.904
**EQ-5D-5L**			
Quality of life visual analogue scale, mean (SD)	70 (19)	73 (19)	0.387
No problems with mobility	29	36	0.815
No problems with self-care	73	66	0.524
No problems with usual activities	40	50	0.519
No pain or discomfort	47	55	0.698
No anxiety or depression	58	71	0.121
**Symptom Status Questionnaire (reported symptoms**)			
Daytime dyspnoea	63	46	**0.035**
Orthopnoea	22	25	0.743
Fatigue or lack of energy	81	61	**0.012**
Chest pain	82	83	0.978
Difficulty sleeping	47	46	0.901
Dizziness or loss of balance	48	35	0.130
Total score, mean (SD)	24.4 (18.4)	22.3 (20.5)	0.503
**EHFScB**			
Total score, mean (SD)	46.5 (21.2)	43.5 (22.2)	0.426
**Responded ‘*do not agree at all*’ on some individual items on EHFScB Scale**			
I weigh myself every day	61	68	0.475
If my shortness of breath increases, I contact my doctor or nurse	48	39	0.418
If my feet or legs become more swollen than usual I contact my doctor or nurse	41	46	0.930
If I gain 2 kg in 1 week I contact my doctor or nurse	72	70	0.937

^a^Unless otherwise stated. EHFSCB = European Heart Failure Self-care Behaviours. EQ-5D-5L = EuroQoL - 5 dimensions - 5 levels. HFpEF = heart failure with preserved ejection fraction. SD = standard deviation.

**Table 4.** Pharmacological treatment by HFpEF diagnosis

Pharmacological agent	HFpEF,*n* = 93, %^a^	Non-HFpEF,*n* = 59, %^a^	*P* value for comparison
Prescribed medications, mean (SD)	8.3 (4.0)	7.8 (3.9)	0.454
>10 medications	37	30	0.398
ACEI	34	37	0.698
ARB	30	32	0.840
ARNI	1	2	0.743
MRA	12	18	0.355
Beta-blockers	48	54	0.475
Calcium channel blockers	32	40	0.315
Loop diuretics	57	51	0.456
Any diuretic	65	61	0.673
Digoxin	16	22	0.334
Statins	58	63	0.552
Aspirin	21	28	0.316
Other antiplatelet	7	5	0.729
Anticoagulation	51	65	0.100
Anticoagulation if AF^b^	96	91	0.409
Antidepressants	16	9	0.232
Anti-anaemia drugs	14	5	0.111
Uric acid-related drugs	19	18	0.813
NSAIDs	2	2	0.845
**Patients with diabetes (*n* = 44**)			
Insulin	25	40	0.307
Biguanides	48	47	0.927
Sulfonylureas	15	20	0.666
SGLT2 inhibitors	7	0	0.535
DPP4 inhibitors	17	20	0.822
**Non-pharmacologic management**			
Attended CR in past	13	16	0.640
Currently attending CR	3	0	0.168

^a^Unless otherwise stated. ^b^ *n* = 48. ACEI = angiotensin-converting enzyme inhibitor. AF = atrial fibrillation. ARB = angiotensin receptor blocker. ARNI = angiotensin receptor-neprilysin inhibitor. CR = cardiac rehabilitation. DPP4 = dipeptidyl peptidase-4. HFpEF = heart failure with preserved ejection fraction. MRA = mineralocorticoid receptor antagonist. NSAIDs = non-steroidal anti-inflammatory drugs. SD = standard deviation. SGLT2 = sodium glucose co-transporter-2.

## Discussion

### Summary

In this cohort of patients recruited mainly from HF registers in primary care (86%), the predominant characteristics of patients with HFpEF were a greater proportion of females, advanced age, and multimorbidity. Significant differences by group were found, as patients with HFpEF had more obesity, pre-frailty or frailty, functional impairment by 6MWD and gait speed, demonstrated lower levels of activity, and had greater likelihood of reporting symptoms, such as dyspnoea and fatigue, than those not confirmed HFpEF.

As might be expected in an older multimorbid sample, patients were taking multiple medications. Sixty-five per cent of patients with HFpEF were taking diuretics, but many presented with signs and symptoms of volume overload such as peripheral oedema. Although few abnormalities were found in laboratory values, HbA1c levels in patients with diabetes indicated that glycaemic control was less than optimal. Findings on the EHFScB indicated that few patients with HFpEF agreed with statements that they regularly performed actions recommended for self-management such as monitoring weight gain or notifying a healthcare provider for signs and symptoms of worsening HF. Patients did not report high levels of depression or anxiety symptoms, and quality-of-life scores were moderately high on both the KCCQ and EQ-5D-5L visual analogue scale.

### Strengths and limitations

This study presents a well-phenotyped cohort of patients with HFpEF recruited mainly from primary care practices in two regions in England, indicating the challenges and problems faced. Recruitment was slow, and likely limited by focusing on patients on practice HF registers, so patients not yet diagnosed with HF or with HF not added to the register were excluded. Future studies may find more patients by searching the practice adult population for those on diuretics or combinations of medications used for HF.^18^ Over half of the eligible sample did not respond to the study invitation, and 58% of those responding declined participation. Information about non-responders or those declining was not collected, but it is plausible that some may have had poorer health or not thought the study was relevant to them. Limited recruitment may have introduced bias in the sample; however, it is notable that the sample was older, multimorbid, functionally impaired, and came from both low and high areas of deprivation. The sample was limited by a high proportion of patients not confirmed as HFpEF. Confirmation of HFpEF was clinically adjudicated using symptoms, signs, NT-proBNP, and echocardiogram data, following European Society of Cardiology (ESC) guidelines criteria.^9^ Future studies may include additional testing to determine diagnosis.

### Comparison with existing literature

The prevalence of comorbidities has been reported to be higher in HFpEF than HFrEF, consistent with the idea that comorbid conditions drive the inflammatory response leading to HFpEF.^16^ The older patients with HFpEF with multiple comorbid conditions, such as obesity, hypertension, diabetes, and kidney disease, has been described as ‘garden variety’ HFpEF, indicating that this is a frequent phenotype encountered in clinical practice.^1^ However, this common phenotype contrasts with HF clinical trials where limited reporting of comorbid conditions and low prevalence of obesity and multimorbidity is usual in recruited patients with HFpEF.^13^ Studies have attempted to delineate patients into distinct phenotypes based on clinical and diagnostic characteristics using patient samples from secondary care centres and clinical trials.^10–12^ Currently there is no agreement on distinct phenogroups, and others have called for simpler designations using single characteristics such as sex, obesity, and atrial fibrillation.^19,20^ This analysis fills a gap in the literature by detailing the characteristics of the prevalent patient who is older and multimorbid with HFpEF in primary care, revealing areas of need in their management.

Multiple studies have shown a greater prevalence of women among populations with HFpEF, although it is unclear whether this is related to higher survival rates of women at older ages, or factors such as the stronger relationship between obesity and incident HFpEF among women compared with men.^21^ Overweight and obesity is highly prevalent in patients with HFpEF (up to 80%), as is frailty.^20,22,23^ A recent analysis of 4605 older patients (mean age 80.3 years) with HFpEF hospitalisation found that 41% had frailty, and that frailty was the most important predictor of re-hospitalisation, and second (after age) for mortality.^23^

Exercise intolerance in HFpEF is owing to both cardiac and peripheral factors, with pro-inflammatory factors, fatty infiltration, and impaired oxidative metabolism leading to decreased muscle strength.^24^ The average 6MWD difference between groups was 65 m. A recent meta-analysis found each 50 m 6MWD reduction was significantly associated with increased risk of all-cause mortality, readmission rates, and combined death or readmission.^25^ Although all patients had low activity levels, the average vector magnitude was lower in those with HFpEF compared with patients without HFpEF, and less than in a UK Biobank sample of patients with HF and in another study of HFpEF.^17,26,27^

Somewhat surprisingly, despite symptoms and limited functional status, quality-of-life scores were moderately high. The developers of the KCCQ define scores from 50 to 74 as fair to good health status, and ≥75 as good to excellent.^28^ The overall score on the EHFScB Scale was low compared with a sample of 1192 patients with either HFpEF or HFrEF (mean score 58.3, mean age 72 years, mean EF 45%), indicating fewer self-care behaviours among the cohort in the present study.^29^

### Implications for practice

The study demonstrates that multimorbidity, polypharmacy, obesity, pre-frailty and frailty, poor physical function, low activity levels, and symptoms are prevalent in patients with HFpEF and present key management challenges. Patients with HFpEF often sit outside of specialist HF services in the UK owing to commissioning restrictions, and primary care therefore takes the lead in managing patients.^8,30^ Current recommendations to manage comorbid conditions and to use diuretics^9^ are not trivial given the number of co-existing conditions, detrimental effects of polypharmacy, and challenges of fluid balance in older adults with renal and functional impairment.

Implications for primary care practice begin with the identification of patients with HFpEF, which likely needs specialist support,^15^ but is important in ensuring appropriate treatment. For example, a decrease in polypharmacy in HFpEF could be enhanced by differentiation of HFpEF from HFrEF. Medications indicated for HFrEF should not be prescribed unless there is another indication (for example, angiotensin-converting enzyme inhibitor for blood pressure control), as they do not exert the same protective effect in HFpEF.^1^ Medication reviews in primary care provide the opportunity to consider the necessity for specific medications.

Over half of the patients in both groups were prescribed diuretics, which often limit their ability and willingness to leave the house. The challenge of managing diuresis is further complicated if patients have incontinence, as reported by almost 20% of patients in the sample. Managing fluid balance also requires consideration of patient behaviours and support to enable patients to monitor signs and symptoms, limit fluid and excessive salt intake if appropriate, and know when to contact a healthcare provider.^9^ Scores on the EHFScB indicated that many patients did not practice behaviours related to self-management. Teaching and supporting self-management should be a component of HF reviews, and all providers need to facilitate this partnership with patients.

Interventions to improve general health status, such as physical activity, dietary enhancement, and management of breathlessness, should be introduced. Exercise training or bespoke cardiac rehabilitation could be developed and commissioned given the evidence of benefit.^27,31^ Home-based targeted rehabilitation, such as in the REACH-HFpEF pilot study,^27^ may improve patient and carer outcomes and be key to ensuring patient participation. The Rehab-HF trial demonstrated that patients who were recently hospitalised and very frail with HF benefit from rehabilitation.^32^

New therapies to treat HFpEF may be added to current medication regimens in the future. Indications from recent studies are that medications such as sodium-glucose co-transporter-2 (SGLT2) inhibitors, spironolactone, and sacubitril with valsartan may be effective, even if in specific subgroups.^1^ The American Heart Association and American College of Cardiology have made a limited recommendation for the use of spironolactone in some patients with HFpEF.^33^

Patients recruited from primary care with confirmed HFpEF demonstrate marked impairment across a range of domains including multimorbidity, functional impairment, and frailty. These findings highlight the need to recognise and record HFpEF as a diagnosis, which would enable clinicians to identify patients and work together to optimise wellbeing through comorbidity management, medication rationalisation, rehabilitation, and self-management.

## Trial registration number

ClinicalTrials. gov (reference number: NCT03617848).
